# Inhibition of Amyloid β Accumulation by Protease-Digested Whitebait (Shirasu) in a Murine Model of Alzheimer’s Disease

**DOI:** 10.3390/foods13182858

**Published:** 2024-09-10

**Authors:** Takahiro Katsuki, Kayako Ogi, Ayaka Kinno, Shingo Kasamatsu, Hideshi Ihara, Hidenobu Sumitani

**Affiliations:** 1 Toyo Institute of Food Technology, 23-2-4, Minami-Hanayashiki, Kawanishi-shi 666-0026, Hyogo, Japan; takahiro_katsuki@shokuken.or.jp (T.K.);; 2 Department of Biological Chemistry, Graduate School of Science, Osaka Metropolitan University, 1-1 Gakuen-cho, Naka-ku, Sakai-shi 599-8531, Osaka, Japan; su23029l@st.omu.ac.jp (A.K.); kasamatsu@omu.ac.jp (S.K.); iharah@omu.ac.jp (H.I.)

**Keywords:** Alzheimer’s disease, β-secretase, whitebait, functional food-derived ingredient

## Abstract

The number of people with dementia is increasing annually worldwide. Alzheimer’s disease (AD), which accounts for the highest percentage of dementia-causing diseases, remains difficult to cure, and prevention of its onset is important. We aimed to discover new AD-preventive ingredients and investigate the inhibitory effects of ten different species of seafood digests prepared by protease treatment on β-secretase 1 (BACE1) activity. Substantial inhibition of BACE1 activity was observed in five species of seafood, and protease-digested whitebait (WPD) showed the highest inhibitory effect among the ten marine samples. We further examined the potential of WPD as an AD preventive component using a familial AD strain (5xFAD) murine model. The intraperitoneal administration of WPD for 28 days substantially decreased the insoluble amyloid β_1–42_ content and the expression of glial fibrillary acidic protein, a marker of astrogliosis, in the cerebral cortex of the 5xFAD mice. These results strongly suggest that WPD is a novel functional food-derived ingredient with preventive effects against AD.

## 1. Introduction

The increase in the number of patients with dementia is an urgent issue globally. The number of people with dementia worldwide is predicted to increase to approximately 153 million by 2050 [1]. Alzheimer’s, Lewy body, vascular, and frontotemporal lobar dementia are well known as the four major causative diseases of dementia, of which Alzheimer’s disease (AD) accounts for over 60% of causative diseases [2]. AD is a cerebral disorder that affects memory, thinking, and behavior. Two types of medicines are used to treat patients, cholinesterase (tacrine, donepezil, rivastigmine, galantamine) and *N*-methyl-d-aspartate receptor inhibitors (memantine), which regulate neurotransmitters and temporarily stabilize cognitive functions; however, both are symptomatic therapies [3]. Lecanemab is an antibody medicine that removes amyloid beta (Aβ) from the brain by binding Aβ soluble protofibrils [4]. In 2023, this medicine was approved by the U.S. Food and Drug Administration for the treatment of AD; however, it is important to administer the drug in the early stages of AD [4]. Thus, AD treatment after onset remains difficult and prevention seems important [3].

The most supported hypothesis about the mechanism of AD onset is Aβ plaque formation, resulting in neuronal cell death and memory disorders [5]. Aβ is a 40–42-residue peptide that is enzymatically produced from amyloid precursor protein (APP) by β-secretase (BACE1) and γ-secretase [6]. BACE1 is an aspartic protease involved in the cleavage of membrane proteins. Especially for APP, BACE1 cleaves the extracellular domain and causes Aβ generation [6]. For AD therapy, BACE1 inhibitors have been actively developed [7], and they were reported to inhibit the accumulation of intracerebral Aβ after intraperitoneal administration in a mouse experiment [8]. Some BACE1 inhibitors have been tested in clinical trials; however, they were discontinued due to insufficient therapeutic efficacy and safety [9,10,11,12]. In particular, it is difficult to recognize the therapeutic effectiveness after AD onset and immediately before the onset of AD [9]. Although BACE1 inhibitors require early administration, it is impractical to routinely administer medicines before the onset of symptoms. In contrast, functional foods, including bioactive components with BACE1 inhibitory effects, can be consumed via daily diets regardless of AD onset, contributing to the prevention of AD development.

Previous studies have demonstrated the BACE1 inhibitory activity of various food-derived components, including polyphenols, such as catechins [13,14,15]; polysaccharides, such as glycosaminoglycans [16,17,18]; bioactive peptides [19,20,21]; and sulfur-containing components, such as sulforaphane [22]. In addition, several BACE1 inhibitory components have been identified in marine products [23], but most of them are derived from seaweeds or bacteria, and there are only a few reports on components derived from fish [23]. Fish are known for their broad-spectrum health-promoting effects; however, to the best of our knowledge, only a small amount of attention has been paid to the inhibitory effects of fish-derived components on BACE1 activity.

Protease digestion is commonly used to prepare protein-derived bioactive peptides and increase the efficiency of the extraction of functional components from foods, including fish [24,25]. However, little is known about the effects and bioactivity of protease digests of fish in the prevention of AD. In this study, we aimed to evaluate the potential of seafood-derived components prepared by protease digestion as functional ingredients for the prevention of AD onset using a fluorescent in vitro BACE1 assay and animal experiments with a familial AD model (5xFAD, [26]) mouse strain.

## 2. Materials and Methods

### 2.1. Protease Digestion of Fish

Ten different types of seafood, sardine (*Sardinops melanostictus*, from Chiba, Japan) squid (*Heterololigo bleekeri*, from Aomori, Japan), horse mackerel (*Decapterus maruadsi*, from Oita, Japan), sea bream (*Pagrus major*, from Hiroshima, Japan), salmon (*Oncorhynchus mykiss*, from Turkey), flounder (*Hippoglossoides dubius*, from Tottori, Japan), shrimp (*Litopenaeus vannamei*, from Indonesia), tuna (*Thunnus albacares*, from Chiba, Japan), mackerel (*Scomber australasicus*, from Kochi, Japan), and boiled whitebait (*Engraulis japonica* fry, from Ehime, Japan), were purchased from a local grocery store. Except for whitebait, the meat parts of the other nine species of seafood were cut into 3–10 mm pieces and used for further protease digestion. The whole whitebait was subjected to protease digestion. Protease solution was prepared by dissolving 9 mg of protease (Amano 3SD, Amano Enzyme, Inc., Nagoya, Japan) in 750 μL of ultrapure water. For protease digestion, seafood samples (900 mg) were mixed with 750 μL of protease solution and incubated at 50 °C for 90 min using a heat block incubator (THB-1, As One Corp., Osaka, Japan). The protease-digested mixtures were then incubated at 95 °C for 10 min to terminate the protease digestion and centrifuged at 7740× *g* at room temperature (approximately 25 °C) for 5 min. The supernatants were filtered using a membrane filter (Watman GD/X 13, 0.45 µm, Cytiva, Tokyo, Japan), lyophilized, and stored at −30 °C until further experiments. Seafood pieces were also prepared in the absence of protease, followed by the same sample procedure, and used as negative controls.

### 2.2. Fluorescent BACE1 Assay

An evaluation of the inhibitory effects of protease-digests of seafood was carried out using a SensoLyte 520 BACE1 Assay Kit (Anaspec, Fremont, CA, USA) according to the manufacturer’s protocol with slight modifications. Lyophilized protease- and non-protease-treated samples were re-dissolved in ultrapure water, and the insoluble fractions were filtered using membrane filters. In a 384-well black plate (Thermo Fisher Scientific, Waltham, MA, USA), 4 μL/well of sample and 16 μL/well of BACE1 enzymatic solution (diluted 1/200 in the assay buffer) were mixed. After a 10 min incubation at 32 °C, the BACE1 enzymatic reaction was initialized by the addition of 20 μL/well of substrate solution (diluted 1/100 in the assay buffer). Fluorescence intensity (excitation wavelength: 490 nm, emission wavelength: 510 nm) was measured at 2 min intervals for 30 min using a microplate reader (CLARIOstar Plus, BMG Labtech, Aylesbury, UK). BACE1 activity was calculated using the following formula:


BACE1 activity = fluorescence intensity at 30 min − fluorescence intensity at 0 min
(1)

### 2.3. Evaluation of BACE1 Activity by Fluorescence HPLC

The inhibitory effects of protease-digested seafood on BACE1 activity were also evaluated using a fluorescent high-performance liquid chromatography (HPLC)-based assay. Human recombinant BACE1 (Cat#931-AS, R&D Systems, Minneapolis, MN, USA) was dissolved in ultrapure water, and fluorescent peptide substrate IV (Mca-SEVNLDAEFRK(DNP)RR-NH_2_; Cat#ES004, R&D Systems) was dissolved in dimethyl sulfoxide (DMSO). They were diluted with 0.1 M sodium acetate buffer (pH 4.0) before evaluation, respectively.

In a 96-well black plate (OptiPlate-96 F, PerkinElmer, Waltham, MA, USA), 50 μL/well of BACE1 enzyme (final concentration 22 nM), 40 μL/well of substrate solution (final concentration 10 µM), and 10 μL/well of BACE1 inhibitor (LY2886721; Cat#S2156, Selleck Chemicals, Houston, TX, USA; final concentration 200 nM) or the same volume of protease-digest samples were mixed and incubated at 37 °C for 60 min in the dark. One hundred microliters of the reaction mixture was collected into a new 1.5 mL tube containing 100 µL of 2.5% trifluoroacetic acid to terminate the enzymatic reaction. After centrifugation at 4 °C and 15,000× *g* for 10 min, the obtained supernatant was subjected to HPLC analysis. Fifty microliters of sample was injected into an HPLC pump consisting of PU-2089 (JASCO, Tokyo, Japan), autosampler (AS-1550; JASCO), and a fluorescent detector (FP 2020 Plus; JASCO), using Mightysil RP-18 GP (75 × 3.0 mm, φ5 µm; Kanto Chemical Co., Inc., Tokyo, Japan) with an isocratic elution of methanol/0.1% formic acid (50/50) with a flow rate of 0.3 mL/min. The fluorescent product cleaved from the substrate peptide was detected by monitoring the fluorescence excitation wavelength at 320 nm and emission wavelength at 405 nm and quantified by a standard curve obtained using 7-methoxycoumarin-3-carboxylic acid.

### 2.4. Preparation of Feed Administration

Lyophilized protease-digested samples were dissolved in 1 mL of ultrapure water and separated using a flash automated purifier (Isolera One; Biotage, Uppsala, Sweden) under the following conditions. Ultrapure water and ethanol (liquid chromatography–mass spectrometry grade, FUJIFILM Wako Pure Chemical Corporation, Osaka, Japan) were used as solvents A and B, respectively. The column was composed of SNAP Ultra 25 g (Biotage) and pre-equilibrated with solvent A. Samples were applied to the column at a flow rate of 10 mL/min and separated using a linear gradient of solvents A and B (0% B for 0–3 min, 0–100% B for 3–26 min, and 100% B for 26–30 min). The fractions of 0–10 min were collected, evaporated with ethanol, and lyophilized. The lyophilized powder was stored at −80 °C until animal testing.

### 2.5. Animal Test

The mouse strain, 5xFAD (B6SJL-Tg6799 strain, [26]) was obtained from The Jackson Laboratory (Bar Harbor, ME, USA), and 9–10-week-old male mice were used. This study was performed in accordance with the Guidelines for Animal Experimentation of Osaka Metropolitan University (Osaka, Japan). All animal experiments were approved by the Animal Ethics Committee of Osaka Prefecture University (protocol code: No. 20-86). The animals were kept at 22–24 °C and with a 12 h light/12 h dark cycle. The animals were fed commercial pellets (CE-2; CLEA Japan Inc., Tokyo, Japan) and provided water ad libitum. The genotype of individual animals was determined using polymerase chain reaction, as recommended by The Jackson Laboratory, using homozygous wild-type (WT) and heterozygous 5xFAD mice.

The mice were divided into four groups: control WT (WT-control, *n* = 8), WT-medicated (WT-IP, *n* = 5), control 5xFAD (5xFAD-control, *n* = 11), and 5xFAD-medicated (5xFAD-IP, *n* = 5). Body weight was measured immediately before administration. Enzymatic digests were administered intraperitoneally at a dose of 500 mg/kg body weight every 2 days for 28 days. Saline was administered to mice in the control group. After the administration period, mice were anesthetized using isoflurane inhalation and euthanized by cervical dislocation. The brain tissue was immediately removed; the cerebral cortex was harvested, frozen in liquid nitrogen, and stored at −80 °C.

### 2.6. Quantification of Insoluble Aβ

Extraction of insoluble Aβ from the cerebral cortex was performed as previously reported [27]. In brief, the cerebral cortex was homogenized in 15-fold volumes of ice-cold 20 mM tris(hydroxymethyl)aminomethane (Tris)-HCl buffer (pH 7.4) containing 150 mM NaCl protease inhibitor cocktail (Nacalai Tesque Inc., Kyoto, Japan) with a Teflon homogenizer for 20 strokes, and centrifuged at 100,000× *g* at 4 °C for 1 h using an ultracentrifuge (Model: CS 120FNX, Eppendorf Himac Technologies Co., Ltd., Ibaraki, Japan) with a S120AT3 rotor (Eppendorf Himac Technologies Co., Ltd.). The resultant supernatant was removed, and the pellet was re-suspended in a 5 M guanidine-HCl (GuHCl, pH 8.0)-containing protease inhibitor cocktail followed by incubation end-over-end overnight at 25 °C. After centrifuging at 23,000× *g* at 4 °C for 30 min, the supernatant was transferred to a new 1.5 mL tube (referred to as GuHCl extract) and stored at −80 °C until further experiments. Aβ content in the GuHCl extract (referred to as the insoluble Aβ fraction) was measured using a Human β amyloid (1–42) enzyme-linked immunosorbent assay (ELISA) Kit (FUJIFILM Wako Pure Chemical Corporation) in accordance with the manufacturer’s protocol. The insoluble Aβ contents were corrected for the protein amount, which was quantified using the Protein Assay BCA Kit (Nacalai Tesque Inc.) with bovine serum albumin as a standard, in accordance with the manufacturer’s protocol.

### 2.7. Western Blotting

Western blotting was performed as previously reported with minor modifications [28]. Cerebral cortex was homogenized in ice-cold 50 mM 4-(2-hydroxyethyl)-1-piperazineethanesulfonic acid buffer (pH 8.0) containing 150 mM NaCl, 10 mM sodium pyrophosphate, 10 mM sodium fluoride, 2% 3-[(3-cholamidopropyl)dimethylammonio]-1-propanesulfonate, 2.5% lithium dodecyl sulfate, 10% glycerol, 2 mM ethylenediaminetetraacetic acid, 2 mM sodium vanadate, 1 mM dithiothreitol, and 1% protease inhibitor cocktail by sonication. After incubation on ice for 30 min, lysates were centrifuged at 23,000× *g* and 4 °C for 30 min. The supernatant was transferred to a new tube, and proteins in tissue lysates were heat-denatured. Proteins (20 μg/lane) were subjected to 12% acrylamide gel electrophoresis, separated by sodium dodecyl sulfate-polyacrylamide gel electrophoresis, and transferred to nitrocellulose membranes. Nitrocellulose membranes were blocked with Blocking One (Nacalai Tesque Inc.), followed by a reaction with an anti-glial fibrillary acidic protein (GFAP) antibody (Cell Signaling Technology, Danvers, MA, USA) and an anti-β-actin antibody (Santa Cruz Biotechnology, Dallas, TX, USA) at 4 °C overnight. After washing three times with TBST (20 mM Tris-HCl, 150 mM NaCl, 0.1% Tween 20, pH 7.6), the membranes were incubated with horseradish peroxidase-conjugated anti-rabbit (Santa Cruz Biotechnology) or anti-mouse (Cytiva) secondary antibodies for 1 h at room temperature. After washing three times with TBST, immunoreactive bands were detected using a chemiluminescence reagent (ImmunoStar LD, Fujifilm Wako Pure Chemical Corporation) and a luminescent image analyzer (LAS-1000 mini, Fujifilm Corp., Tokyo, Japan). Band intensities were quantified using analysis software (Multigauge, Fujifilm Corp.).

### 2.8. Statistical Analyses

All experiments were performed at least three times, and values for individual experiments are presented as mean ± standard deviation (SD) or error (SE). Statistical significance was determined using Student’s unpaired *t*-test and the Tukey–Kramer test using the GraphPad Prism 8.1.2 software (GraphPad, Inc., La Jolla, CA, USA); *p* < 0.05 was considered significant.

## 3. Results

### 3.1. Screening of BACE1 Inhibitory Activity in Vitro

**Figure 1:**
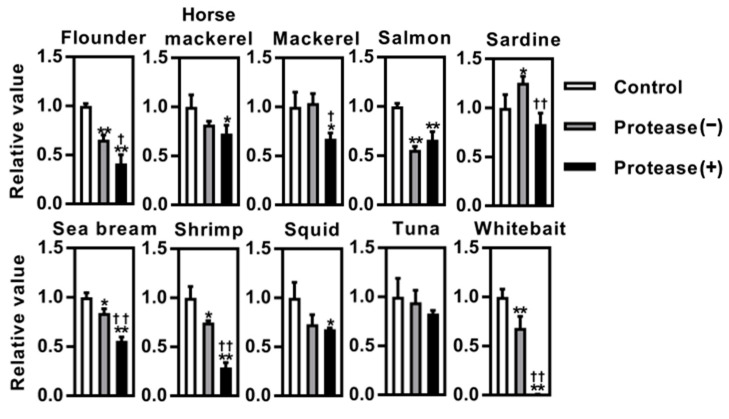
β-secretase 1 (BACE1) inhibitory activity of protease-digested derivatives of seafood. Each lyophilized protease-digested fish powder was dissolved in ultrapure water to 500 µg/mL (final assay concentration, 50 µg/mL). Non-protease-digested samples were dissolved in the same amount of ultrapure water. Ultrapure water was used in the control. White, gray, and black bars represent the control, non-protease-digested, and protease-digested samples, respectively. Data represent the mean ± standard deviation (*n* = 3 each); * *p* < 0.05, ** *p* < 0.01 versus the control, ^†^ *p* < 0.05, ^††^ *p* < 0.01 versus the protease (−).

Protease-treated digests were prepared from ten different species of seafood, including sardine, squid, horse mackerel, sea bream, salmon, flounder, shrimp, tuna, mackerel, and boiled whitebait, and their inhibitory effects on BACE1 activity were evaluated (Figure 1). Compared to the control group, BACE1 activity was substantially inhibited in the presence of protease-digested seafood derivatives (squid, horse mackerel, sea bream, salmon, flounder, shrimp, mackerel, and boiled whitebait), except in the presence of sardine and tuna. Moreover, we confirmed protease digest-dependent BACE1 inhibition in five species of seafood (sea bream, flounder, shrimp, mackerel, and boiled whitebait), and the most remarkable inhibition was observed in the presence of protease-digested whitebait (WPD), which almost completely blocked BACE1 activity (less than 1% activity compared with the control). Based on these results, we further evaluated the potential of WPD.

**Figure 2:**
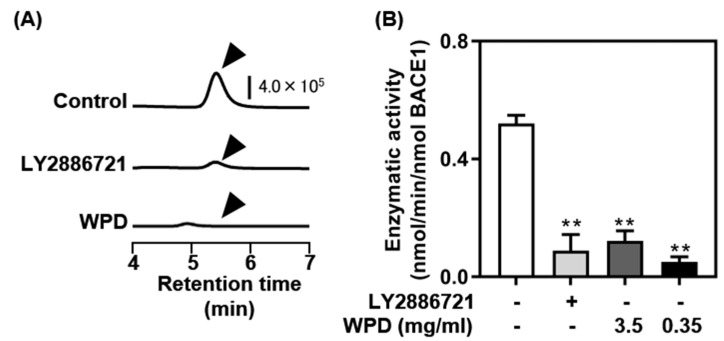
Confirmation of β-secretase 1 (BACE1) inhibitory effects of protease-digested whitebait (WPD) by fluorescent high-performance liquid chromatography (HPLC): (**A**) HPLC chromatograms of the BACE1 enzymatic reaction in the absence (control, **top**) or the presence of LY2886721 (**middle**) or WPD (**bottom**). (**B**) BACE1 enzymatic activities were calculated using a standard curve of 7MCA. Data represent mean ± standard deviation (*n* = 3 each); ** *p* < 0.01 versus the control.

We performed a different assay to support the effect of WPD on BACE1 inhibitory activity using a fluorescent HPLC system (Figure 2). A strong signal of the BACE1-dependent enzymatic reaction product was observed at a retention time of 5.5 min (Figure 2A, top). Only a modest signal was detected in the presence of the well-established BACE1 inhibitor LY2886721 (Figure 2A, middle). Similarly, the signal disappeared almost completely in the presence of WPD (Figure 2A, bottom). Calculation of the amount of the BACE1-dependent enzymatic reaction product using a standard curve of 7MCA demonstrated that WPD strongly inhibited BACE1 activity, which was similar to that of LY2886721 (Figure 2B).

### 3.2. Evaluation of Effects of WPD Administration on 5xFAD Mice

**Figure 3:**
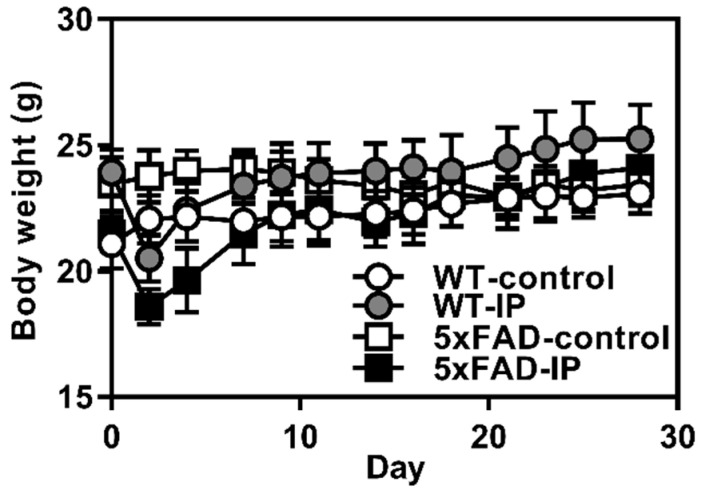
Effects of protease-digested whitebait (WPD) administration on body weight of 5xFAD mice. WPD was administered intraperitoneally every 2 days for 28 days, and the body weight was measured immediately before administration. The white circle shows the WT control group (*n* = 8), the gray circle shows the WT WPD group (day 0: *n* = 11, day 2: *n* = 10, day 4: *n* = 7, days 7–28: *n* = 5), the white square shows the 5xFAD control group (*n* = 11), and the black square shows the 5xFAD WPD group (*n* = 5).

To evaluate the potential of WPD in the prevention of AD in vivo, we performed animal experiments using AD model 5xFAD-strain mice with the intraperitoneal administration of WPD. A slight and transient decrease in body weight was observed in the WPD-administered groups (both WT-IP and FAD-IP); there was no marked change in body weight among any of the four groups on the final day of the administration period (Figure 3). However, six mice died during days 0–7 in the 5xFAD-IP group.

**Figure 4:**
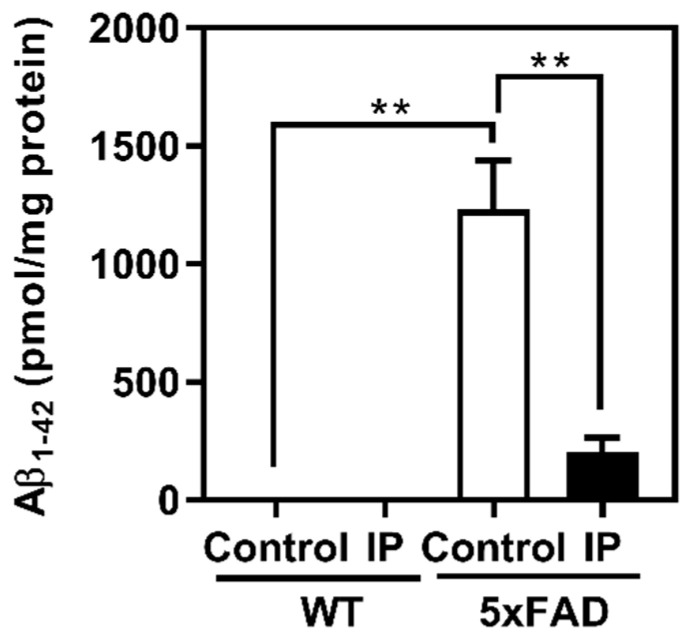
Decrease in the insoluble Aβ_1–42_ content in the cerebral cortex after protease-digested whitebait administration. The insoluble Aβ_1–42_ content in the cerebral cortex was quantified using an enzyme-linked immunosorbent assay. WT control group (*n* = 8), WT WPD group (*n* = 5), 5xFAD control group (*n* = 11), and 5xFAD WPD group (*n* = 5); ** *p* < 0.01.

At the end of the administration period, the cerebral cortex was harvested and the insoluble Aβ_1–42_ content was quantified using ELISA (Figure 4). The results indicated that the insoluble Aβ_1–42_ content in the control 5xFAD mice (1231.9 ± 198.3 pmol/mg protein) was substantially higher than that in the control WT, consistent with previous reports [26]; the results suggest that amyloid plaque was formed in the cerebral cortex of 5xFAD mice. In contrast, a marked decrease in the insoluble Aβ_1–42_ content was observed in the WPD-administered 5xFAD mice (206.2 ± 53.11 pmol/mg protein) compared with control 5xFAD mice. In WT mice, insoluble Aβ_1–42_ was not observed regardless of WPD administration.

### 3.3. Analysis of Astrogliosis

**Figure 5:**
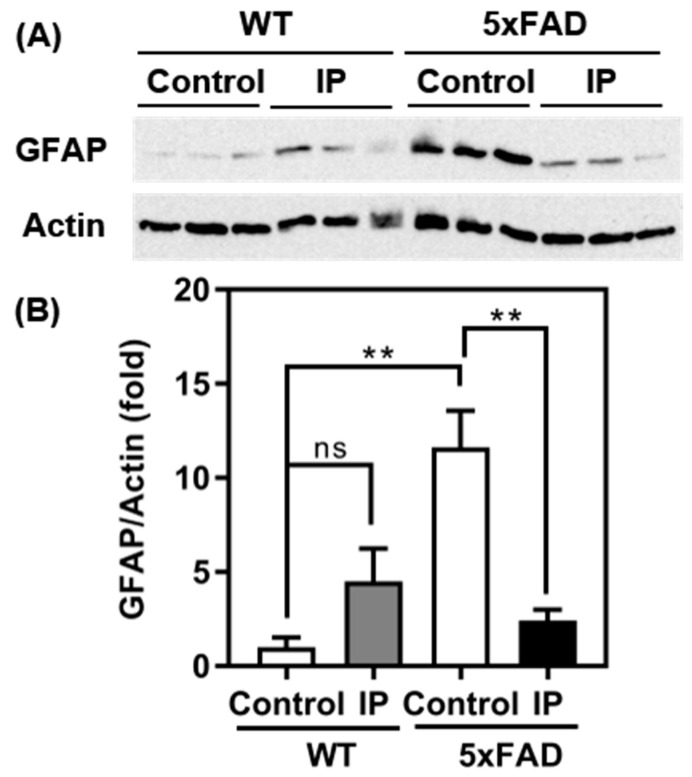
Effect of protease-digested whitebait (WPD) administration on GFAP expression in the cerebral cortex of mice. GFAP expression in the cerebral cortex was detected using Western blotting: (**A**) Representative image of Western blotting using anti-GFAP (**upper**) and anti-β-actin (**lower**) antibodies. (**B**) Relative band intensity of GFAP to β-actin. WT control (*n* = 3), WT WPD (*n* = 3), 5xFAD control (*n* = 3), and 5xFAD WPD group (*n* = 3); ** *p* < 0.01; ns, not significant.

Aggregated forms of Aβ are known to cause aberrant activation of astrocytes (astrogliosis) in the brain [29,30]. Therefore, we examined the effects of WPD administration on the expression of GFAP, a marker of astrogliosis, using Western blotting. A marked increase in GFAP expression was observed in control 5xFAD mice compared to WT mice (Figure 5). In contrast, the WPD-administered 5xFAD mice showed a marked decrease in GFAP expression in the cerebral cortex compared with that in control 5xFAD mice. In WT mice, no difference in GFAP expression was observed upon WPD administration.

## 4. Discussion

In this study, we examined the potential of ten different seafood species, including sardine, squid, horse mackerel, sea bream, salmon, flounder, shrimp, tuna, mackerel, and boiled whitebait, using both in vitro and in vivo experiments. Using the in vitro BACE1 assay, a marked inhibitory effect was confirmed in 8 of the 10 species. Furthermore, in sea bream, flounder, shrimp, mackerel, and boiled whitebait, the inhibitory effects were enhanced by protease pretreatment (Figure 1). Consistent with the present results, several previous studies have reported that muscle protein hydrolysates of marine animals, including shrimp [21] and a typical herbivorous gastropod sea hare (*Aplysia kurodai*) [31], exhibit an inhibitory effect on BACE1. In the current study, the results obtained from two different in vitro BACE1 assays revealed that protease-digested derivatives of boiled whitebait (WPD) exhibited the most remarkable effects on BACE1 inhibition (Figure 1 and Figure 2). To the best of our knowledge, this study provides the first evidence that WPD has strong potential to block BACE1 activity.

The results obtained from the animal experiments using 5xFAD mice demonstrated that intraperitoneal administration of WPD strongly suppressed not only the accumulation of insoluble Aβ, but also the expression of GFAP protein, a marker of astrogliosis, in the cerebral cortex (Figure 4 and Figure 5). Astrogliosis is reportedly caused by the accumulation of Aβ [30,31]; thus, the suppression of astrogliosis observed in the WPD-administered 5xFAD group may be because the WPD administration effectively inhibits BACE1 activity in vivo, resulting in the suppression of Aβ accumulation and downstream astrogliosis.

Whitebait is a collective term for the immature fry of fish, including the Japanese anchovy and sardine. WPD potently inhibited BACE1 activity, whereas no such effect was observed in sardines (Figure 1). Based on these results, we predicted two possibilities. First, the bioactive substances contained in whitebait may change during fish development and aging. Peptide fragments prepared by the enzymatic degradation of several foods exhibit BACE1 inhibitory activity [19,20,21,31] and improve cognitive function in vivo [32,33]. Thus, whitebait may be abundant in these precursor proteins. In the future, mass spectrometry-based analyses will facilitate the identification of peptides present in WPD and the investigation of their pharmacological effects in vitro and in vivo.

Another possibility is that while WPD was prepared by protease digestion of whole shirasu, only the meat was used for protease digestion of sardines, suggesting that bioactive substances with BACE1 inhibitory activity may exist in parts other than the meat. In relation to the latter possibility, it has been reported that chondroitin sulfate, a sulfated glycosaminoglycan that is abundantly present in fish waste [34], has BACE1 inhibitory activity [17,35,36]. Therefore, although further studies are required to identify bioactive substances with BACE1 inhibitory activity and to analyze their pharmacological effects in vivo, functional ingredients with BACE1 inhibitory activity produced from fish waste could lead to the effective utilization of unused resources.

In the animal study, transient weight loss was observed in the early phase of treatment, and six mice died during the administration period in the 5xFAD-IP group. Since the sample used in this animal study was a crude purified product of protease-digested whitebait, various substances other than the active ingredient coexisted, and these ingredients may have contributed to the toxicity. In the future, identification and isolation of the active ingredients in WPD will facilitate a detailed evaluation of the pharmacological activity of the active ingredients in whitebait under low-toxicity dosing conditions.

Several previous studies using 5xFAD mice reported that accumulation of Aβ in the brain was suppressed to approximately half of that in the control group by the administration of natural compounds (i.e., hop-derived iso-α-acids, *Inula britannica*-derived 1,6-O,O-diacetylbritannilactone, whey-derived β-lactolin peptide, and a Chinese natural medicine, Qi-fu-yi) [32,37,38,39]. In the present study, the insoluble Aβ_1–42_ content in the cerebral cortex of WPD-administered 5xFAD mice markedly decreased to one-sixth of that in control 5xFAD mice, suggesting that WPD can be a functional ingredient derived from food with an AD-prevention effect. In this study, WPD was administered intraperitoneally to mice to eliminate the effects of digestion and absorption in vivo. Therefore, it is essential to evaluate the potential of WPD as a food ingredient in future studies using oral animal experiments.

Although Aβ accumulation and GFAP protein expression were evaluated in this study, cognitive assessment was not performed. 5xFAD mice are reportedly impaired in the Y-maze test at 16–20 weeks [26,40]. The current study terminated administration at 13–14 weeks, which was too early to observe impaired cognitive function. This study aimed to prevent AD onset, and administration was initiated at 9–10 weeks, when Aβ accumulation began. The study was terminated at 13–14 weeks because it was difficult to prepare sufficient amounts of the purified product until the age of impaired cognitive function. Because cognitive assessment is a key factor in AD studies, we plan to extend the duration of this study in the future.

## 5. Conclusions

In this study, BACE1 activity was evaluated in ten protease-digested fishes. Five protease-digested fishes suppressed BACE1 activity, of which WPD showed the most remarkable inhibition of BACE1. To evaluate the in vivo effects of WPD, WPD was administered to 5xFAD mice, and the accumulation of Aβ and the expression of GFAP protein in the cerebral cortex were found to be suppressed. Thus, WPD was effective in preventing AD both in vitro and in vivo. Moreover, compared with previous studies on natural compounds, WPD may have more powerful AD-preventive effects. Based on these results, WPD may be a novel and effective food for AD prevention.

## Data Availability

The original contributions presented in the study are included in the article, further inquiries can be directed to the corresponding author.
